# New vessels detected on wide-field imaging compared to two-field and seven-field imaging: implications for diabetic retinopathy screening image analysis

**DOI:** 10.1136/bjophthalmol-2015-306719

**Published:** 2015-08-13

**Authors:** Stephen James Talks, Vina Manjunath, David H W Steel, Tunde Peto, Roy Taylor

**Affiliations:** 1 Newcastle Eye Centre, Royal Victoria Infirmary, Newcastle upon Tyne, UK; 2 Sunderland Eye Infirmary, Sunderland and Institute of Genetic Medicine, Newcastle upon Tyne, UK; 3 NIHR Biomedical Research Centre at Moorfields Eye Hospital NHS Foundation Trust and UCL Institute of Ophthalmology, London, UK; 4 Magnetic renounce centre, Campus for ageing and vitality, Newcastle University, Newcastle upon Tyne, UK

**Keywords:** Diagnostic tests/Investigation, Imaging, Neovascularisation, Retina

## Abstract

**Introduction:**

Wide-field retinal imaging (Optomap), used for detecting diabetic retinopathy (DR), has been shown to compare well with seven-field early treatment diabetic retinopathy study (ETDRS) photographs. An Optomap 200° image covers 80% of the retinal surface, compared with the standard seven-field, 30° images, covering 30% of the retinal surface. In England, DR screening is performed by grading two, 45° images per eye, by the DR screening service (DRSS).

**Purpose:**

To assess how often retinal new vessels (NVs) are observed on Optomap imaging, outside the DRSS two fields and standard seven-field photography, in a cohort of patients referred by the DRSS.

**Method:**

A consecutive series of treatment naïve patients with DR, referred from DRSS with pre-proliferative or proliferative DR or diabetic maculopathy, were imaged with Optomap colour images, within 3 months of DRSS referral. The incidence and distribution of NVs were recorded in relation to two-field and seven-field areas.

**Results:**

NVs were found in 102 of 1562 treatment naïve eyes (6.5%) of 781 patients. Of these, 72 were referred from DRSS as having NVs, but an additional 30 eyes (29% of NVs detected) from 25 patients were referred with a lesser degree of DR. In 25 of the 30 eyes without NVs reported on referral, NVs were located outside the standard two fields taken at DRSS, and in 12, NVs were outside the area covered on seven-field imaging (11.7% of eyes with NVs).

**Conclusions:**

Wide-field imaging with Optomap detected approximately 30% more NVs than standard two-field imaging in patients referred from a UK DRSS.

## Introduction

Wide-field retinal imaging (Optomap), used for detecting diabetic retinopathy (DR), has been shown to compare well with seven-field early treatment diabetic retinopathy study (ETDRS) photographs.^1–3^ As it provides a wider field of view, it would not be surprising that more DR is seen; however, it is thought that most potentially sight-threatening pathology occurs between the posterior pole and retinal mid-periphery. Silva *et al*^4^ reported that 10% of a cohort of 206 eyes were given a higher DR grade on Optomap images compared to seven-field images, predominantly due to the finding of more haemorrhages per quadrant. In his study, the patients were chosen from a tertiary eye clinic to represent a range of DR severity, and so, incidence rates of previously not recorded findings in a population referred from the community could not be assessed. Grading is based on the ETDRS studies that related retinal findings to the likelihood of progression of the retinopathy and is based on seven-field colour imaging. It is still unknown how often more severe DR changes are found outside the standard seven-field, in particular new vessel (NV) formation. In the English DR screening service (DRSS) two images with nominal 45° fields are taken per eye, one centred on the fovea and the other on the disc. This is said to have a sensitivity of 80.2% and specificity of 92.9% for detecting referable DR compared to slit lamp biomicroscopy.^5^ In this study, we aimed to assess how often NVs were seen with wide-field Optomap imaging when compared to the areas covered by DRSS's two-field and standard seven-field photography, in a cohort of patients referred from a DRSS.

## Method

A consecutive series of treatment naïve patients, referred from two DRSS in England, were imaged with Optomap colour images, within 3 months of referral. Referral from DRSS occurs if ‘referable’ DR is detected on analysis of two standard 45° photographs. At DRSS images are graded for the level of DR, and diabetic maculopathy (DMac): no DR is denoted as R0; mild DR as R1; pre-proliferative DR as R2; proliferative DR as R3. Potentially clinically significant DMac is represented by the M1 grade. R2, R3 and M1 are then subsequently referred to hospital eye services.

**Figure 1:**
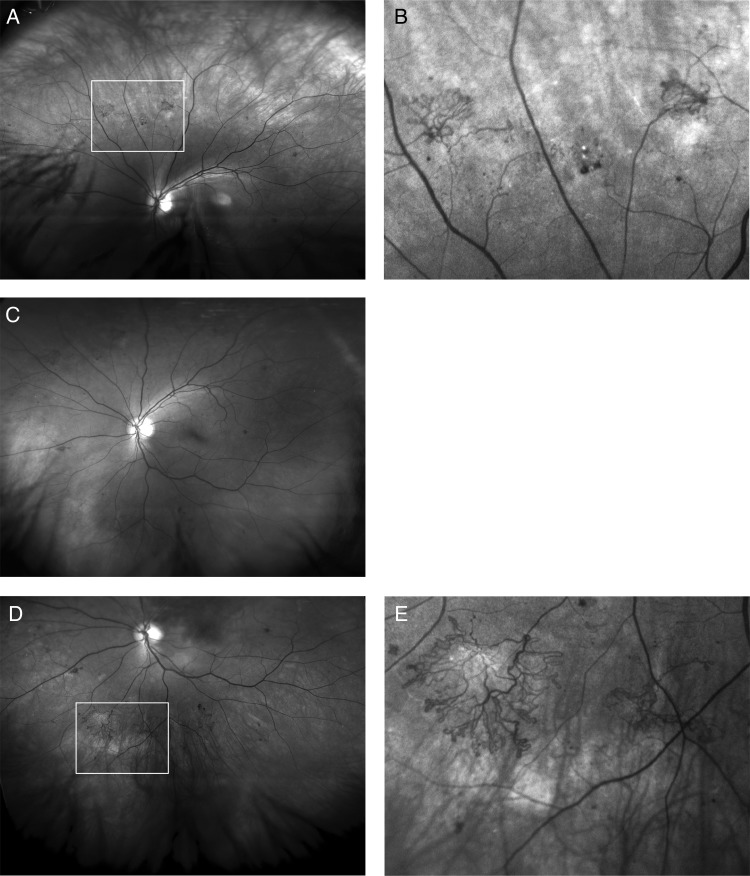
Red free Optomap images of a left eye of a diabetic referred with maculopathy in the other eye, (R1, M1), the left eye being referred as, (R1, M0). (A) Up-steered, showing new vessels, (B) with zoom, (C) straight ahead showing new vessels outside two fields and on the edge of the standard seven-field images, (D) down steered, showing new vessels and (E) better seen with on zoom.

At the hospital eye clinic, certified medical photographers took three wide-field Optomap images per eye after mydriasis, using the Optomap P2000 scanning laser ophthalmoscope; straight-ahead and up and down, with eye steering, which involves the patient following a fixation target (figure 1).

**Figure 2:**
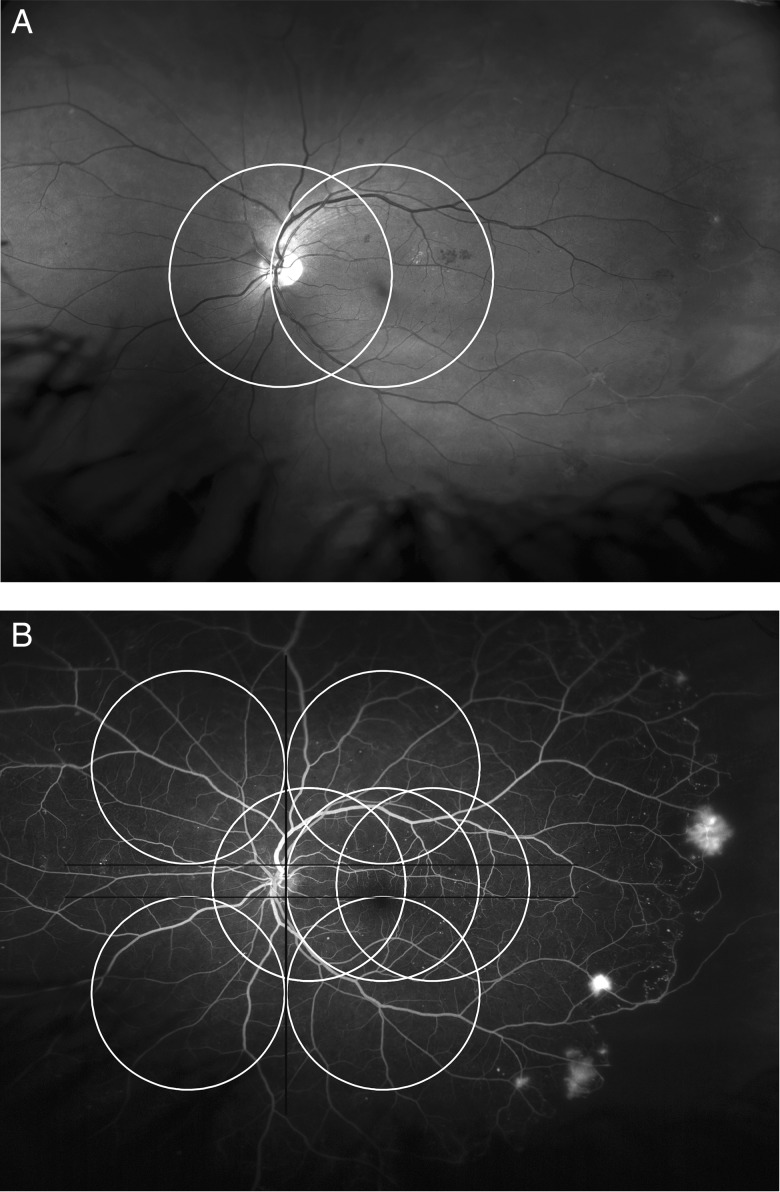
Red free Optomap and fundus fluorescein angiogram of the left eye of a diabetic referred due to maculopathy, (R1, M1 right; R1, M1 left), showing new vessels outside standard two-field (A); and seven-field (B) in the left eye.

The images were then graded by an independent reading centre and the number of eyes with NVs, R3, recorded. The R3 images were then further assessed to map the distribution of NVs in relation to two-field and seven-field standard images using a standard field map (figure 2). If there was more than one area of NVs and any were located inside either the two-field or seven-field areas, then they were counted as being detected by that method. In a few cases, where the distinction between haemorrhage, intra-retinal microvascular abnormalities (IRMA) and small NVs was uncertain, a fundus fluorescein angiogram (FFA) was performed, at the examiners discretion, on a second visit.

The images were reviewed using the proprietary image review software (Optos V2 Vantage Dx Review V.2.5.0.135; Optos, Dunfermline, UK). Grading for each wide-field image involved viewing the colour composite, green-wavelength and red-wavelength images using all the available image enhancement tools, including localised optimisation and magnification. However, true NVs were recorded only if the NV was present on FFA where available and also where the NV remained clearly visible on the original image once spotted on the manipulated image, where appropriate.

## Results

Independent assessment of the wide-field imaging found NVs in 102 of 1562 treatment naïve eyes (6.5%) of 781 patients referred from DRSS. Of these, 72 were referred from DRSS as having NVs, but 30 eyes (29% of NVs detected) from 25 patients were referred with a lesser degree of DR: 14 were referred as R2 and 16 as R1. In these eyes, 18 had been referred with Dmac, (M1), the other 12 were graded as M0. The fellow eyes of these patients were graded as R3 (proliferative DR) in three cases, R2 in eight and R1 in nine. In nine cases both eyes were referred with R1, having been referred because of DMac.

FFAs were requested in 31 patients where the examiner had some uncertainty between R2 and R3. In 14 of these cases, the examiner had diagnosed NVs and this was confirmed in 10 cases but not found in 4 cases and in 17 cases the examiner thought they were seeing, R2 changes, this was confirmed in 9 cases, but NVs were found in 8 cases.

In 25 of the 30 eyes, in which NVs were not reported on the two DRSS images, the NVs were found outside the standard two-field and in 12 of these outside seven-field (11.7% of eyes with NVs); three were just within the field of view of the two fields but had been referred as R2 with IRMA. Two had very small disc NVs but also had NVs beyond two fields. Images from 23 eyes of the referred 1562 (1.4%) were deemed ungradable on Optomap due to poor image quality.

Figure 1 shows NVs outside two fields and the benefit of using up and down eye steering. Figure 2 shows NVs outside seven fields confirmed on FFA.

## Discussion

ETDRS seven-field colour imaging is still considered the gold standard for assessing DR; however, it is hard both on the patient and on the photographer to use this protocol in everyday clinical practice. In the Eurodiab paper justifying the use of 45° imaging criteria only 48 eyes were compared.^6^ The findings supported the use of two-field imaging as a practical method, with the agreement for correct DR between several examiners, ranging between 28 and 43 of the 48 eyes, mean of 37 eyes. The kappa for interobserver and intraobserver comparisons was good at 0.83 and 0.85, respectively. Two-field imaging, where approximately 80% of patients are imaged yearly, using this protocol meets the appropriate sensitivity and specificity required for a screening programme and was therefore rolled out with scale, as shown by the England DRSS.^7^

In one study, seven-field ETDRS stereo images were ungradable by strict grading criteria in 31.6% and in 15.3% with a more lenient approach.^5^ The same paper reported good agreement for detecting the difference between referable and non-referable retinopathy between slit lamp biomicroscopy, 2×45° field and 7×30° field photography. However, there was only agreement on finding proliferative DR in 51/88 (58%) patients when comparing seven-field ETDRS stereo images with slit lamp examination. It is not clear how many had already had laser which may have lead to confusion on definitions between active or inactive NVs. In two cases, the clinician found NVs outside seven-field. For the comparison of two-field to seven-field only correlations between detecting referable from non-referable DR were presented.

Our study shows that on two-field DRSS imaging there is only a small risk of missing NVs, 30/1562 (1.9%), but these represented 29% of the total number of eyes graded as having NVs. The NVs were found outside even the seven-field area in 11.7%. This is a higher rate than previously reported. In a study of 206 eyes of 103 patients, 10% were given a more severe DR grade with wide-field imaging, using one image per eye.^4^ In relation to our findings, 46 had NVs, but only two of these were found outside the seven-field area (4% of NVs). Our study population was much larger and represents a consecutive series referred from DRSS, rather than a group from a highly specialised clinic.

A study using wide-field FFA on 118 patients found a total of 22 eyes (10%) had pathology visible only outside a simulated seven-field boundary. Of those eyes, 13 had peripheral retinal non-perfusion (8%) and 9 of 54 cases (17% of NVs) had peripheral NV outside seven-field. While using a different technique for identification of cases with NVs, this study draws a similar conclusion to ours on the relative proportion of NVs found outside seven-field.^8^ In the cases where we did use FFA some changes were made in the grading and eight additional cases of NVs were found.

One factor that may have led us to detect this rate of NVs was the use of three images per eye, using eye steering, as less pathology is likely to be missed due to defocus or masking from eyelashes.^9^ The Optos camera can take a 200° image, but the resolution is best in a central band between the two arcades. The focus for the top and bottom areas of the retina is better by taking the image with the patient looking up and down.

Looking at three images per eye takes extra time compared to one. Montage software is being developed to merge three pictures, which will help with analysis, but is not commercially available yet.

A study comparing wide-field photographs, taken with undilated and dilated pupils, found that this did not statistically change the agreement with seven-field imaging, but reduced the ungradable rate from 4.5% to 0%.^4^ We had an ungradable rate of 1.4% using dilation and three images per eye.

Our patients with NVs not detected on DRSS images were not ‘missed’ cases, as they were correctly referred for further medical assessment. All registered patients with diabetes are offered annual DRSS photography in England and this has led to fewer patients being referred from DRSS with severe NVs, and so, our incidence figures of more peripheral pathology may be higher than in unscreened populations. It is possible that if patients had small NVs outside the two-field images they would have been eventually referred as more posterior pathology developed.

This study also does not clarify how much risk there is in missing peripheral NVs, as they were not detected as a result of a patient presenting with the complications of proliferative DR, rather as a result of imaging a cohort of patients. However, if NVs are missed on DRSS images, and the patient is referred because of Dmac, appropriate management depends on the clinician detecting these NVs, which may not occur in a busy streamlined macular service. We would therefore advocate the use of steered wide-field images in ophthalmology clinics, and in the future hope that automated software can be developed to allow for fast, reliable and valid identification of abnormal vessels.
